# Carework as resistance: How incarcerated women care for each other to survive carcerality amid a global pandemic

**DOI:** 10.1111/maq.70034

**Published:** 2025-11-11

**Authors:** Esther Melton, Leslie Riddle, Jennifer Elyse James

**Affiliations:** 1 School of Medicine, University of California, San Francisco, San Francisco, California, USA; 2 Department of Humanities and Social Sciences, School of Medicine, University of California, San Francisco, San Francisco, California, USA; 3 Institute for Health and Aging, School of Nursing, University of California, San Francisco, San Francisco, California, USA

**Keywords:** carework, COVID-19, incarceration

## Abstract

The COVID-19 pandemic was a crisis in prisons and jails, with some of the largest outbreaks in the United States happening inside carceral facilities. In the absence of structural interventions to protect them, people inside prisons engaged in various forms of carework to support one another and to draw attention to the horrific conditions. We conducted interviews and focus groups with people who were incarcerated during the COVID-19 pandemic; healthcare workers in prisons and jails; and advocates and organizers supporting people in carceral settings. Interviews were triangulated with field notes from ethnographic observations of medical and legal advocacy efforts during the pandemic. We argue that the carework performed by people incarcerated is a key form of invisibilized labor and resistance within carceral settings. We describe how this labor is both relied on for prisons to function, particularly during a public health emergency, and an under-recognized element of abolitionist organizing in women’s prisons.

## INTRODUCTION

*I remember we was watching TV and they had a breaking news about this virus and they said it’s hitting California. So, at first start we were scared because we didn’t know if it got into prison, what would happen to us. We didn’t know if the COs* [Corrections Officer] *were bringing it in on their own. We didn’t know anything. We just knew we had to keep ourselves protected.* – Shay Shay, 48, incarcerated during the COVID-19 pandemic

In March of 2020, as the whispers about a mysterious illness in China became a cacophony of panic and later a wave of sudden intense policy shifts, many of us were fearful and uncertain. As shelter-in-place orders took hold, schools and offices closed, and the numbers of infections began to rise. Many across the United States and around the world spoke of feeling trapped, *imprisoned* in our homes.

While those of us on the outside feared close contact with the virus, people in prisons and jails were quite literally locked in, forced to breathe each other’s air and hope they were not the next victim of the disease. But with people moving in and out of their facilities each day, they unwittingly were exposed to potential vectors of a disease from which they had few tools to protect themselves. In January 2020, there were more than 122,000 people incarcerated in California (Harris, 2021). State prisons were running at more than 130% capacity, with many jails facing similar overcrowding. Everyone who lived in, worked in, or cared for someone in a prison or jail was scared. As one correctional medicine physician reflected, “I thought… we were going to have body bags in the hallway. Like I just thought it was going to be a huge disaster.”

The looming threat of the pandemic was crushing, and it became apparent that the question wasn’t *if* COVID would come to California but *when*. People living in prisons were experiencing the same dread and anticipation as those outside. They were seeing the same nightly news reports and hearing about changes in the world from their loved ones. What was already apparent to those inside was that they could not count on their institution for safety, and they would need to find creative ways to continue to care for each other.

In response to this fear, our research team began, almost unintentionally in the early days of the pandemic, an 18-month virtual and digital ethnography of California’s response to the crisis of the COVID-19 pandemic in prisons and jails. We were already engaged in research on health and illness inside prisons, and COVID-19 became the most, at times the only, pressing concern. The ethnography started slowly with screenshots of media stories and social media posts as cases began to pop up in prisons and jails across the states. We read and indexed reports and recommendations from experts who sounded the alarm about the catastrophic consequences that would arise from delayed action. At the same time, policies put in place across the country, intended to stop the spread of the virus, had the additional effect of altering the spread of information; advocates, loved ones, and volunteers were suddenly prohibited from entering carceral facilities. New coalitions and working groups formed to share any information learned from broader networks. Our team began engaging in participant observations of virtual rallies, organizing meetings, and strategy sessions held by these newly formed coalitions. As we experienced the pandemic unfolding, we were in community with organizers and advocates adapting to new models of organizing while responding to an ongoing and constantly evolving emergency.

In June 2020, the San Francisco Chronicle published the “pandemic diaries” of several people incarcerated in California’s women’s prisons, documenting the experience of COVID behind bars (Fagone, 2020). One woman, April Harris, wrote about being quarantined in isolation, unable to leave her cell for days at a time. She wrote, “Someone is yelling for help over and over and over. No one is coming. This one is scaring me. She keeps screaming. It’s piercing.” Others witnessed crises and trauma, heard suicide attempts, and even saw a woman set fire to her mattress, all while unable to intervene. These diaries produced a stunning account of the trauma of watching friends and loved ones inside suffer while being unable to intervene or offer support. While an outpouring of data and publications brought attention to narratives of fear (Craig et al., 2023), acknowledgements of heightened vulnerability (Hawks et al., 2020), and the need for a robust public health response (Puglisi et al., 2020; Saloner et al., 2023), these diaries were one of the first times our team understood how the pandemic disrupted *carework* provided by and for incarcerated people, and forced people inside to find new ways to continue this life-saving work.

The realities of life behind the razor wires and steel doors of prisons are intentionally hidden, invisible under the guise of safety and security, but with the intended consequences of limiting accountability and obfuscating the violence of the system. April Harris and the others who spoke out did so at risk of retaliation, in the hopes that policies may shift, and others may not endure what they suffered (Song et al., 2023). Sharing their stories during an ongoing pandemic was a stunning act of bravery, resistance, and carework, which they did when the forced separation of quarantine prevented them from physically and emotionally reaching out to each other.

### Conceptualizing carework in prison

In anthropological literature, carework is often conceptualized in terms of its relationship to kinship, neoliberalism, feminist scholarship, and transnational migration of caregivers (Thelen & Coe, 2019). Thelen and Coe argue that the “generative or productive force of care,” which “mobilizes social, material, and labor resources,” helps to also define and shape political belonging. We define carework in the prison context as the emotional, material, informational, and advocacy-based support incarcerated people provide for one another as a “generative force” that resists dehumanization. We also draw on Callahan and Paradise’s (2017) articulation of “fierce care” as a “commitment and practice that continues to suture community beyond just one extended family,” which “exposes the privatized and militarized violence of capitalism,” and “reveals the convivial practices and related tools of care that are outside of the rhythms of capitalist reproduction.” Fierce care refuses disposability and extraction imposed on marginalized people under capitalism. We see carework in the prison context as a necessary, invisible labor that resists a violent state apparatus. In this paper, we describe the role of carework in achieving health, safety, and community in the carceral setting. We reflect on Joy James’ (2019) Captive Maternal to ground our identification of incarcerated people as provisioners of fierce care—care that extends beyond familial care and refuses disposability of marginalized people under capitalist societies. This freedom-seeking carework performed by incarcerated people reflects centuries of resistance pushed forward by individuals forced into captivity, extracted from, and readily disposed of by the state. Moreover, COVID-19 demanded an evolution in this care, necessitating a communal commitment to health and well-being among incarcerated people, which is antithetical to the carceral system. We attempt to explain not only the longstanding role of carework in carceral settings, but also to highlight its revolutionary capacity as it adapted to the changing prison landscape surrounding COVID.

## METHODOLOGY

The findings described here stem from a larger research project on the health of currently and formerly incarcerated older adults during the COVID-19 pandemic. This included 18 months of digital ethnography of legal and medical advocacy work (Forberg & Schilt, 2023), three virtual focus groups with organizers and activists, interviews with 10 women who lived in prisons and jails during the pandemic, and interviews with 10 healthcare providers working in prisons and jails during the pandemic. We engaged in participant observation of virtual public meetings, rallies, and organizing meetings about the pandemic in California prisons to follow advocacy efforts and state responses. We also documented and analyzed news, social media, reports from public health departments, legal filings, and other digital records as they were made available. In this paper, we draw primarily from our analysis of data collected directly from advocates and organizers involved in supporting incarcerated people during the pandemic.

The advocates and organizers in our focus groups represented a variety of grassroots organizations and coalitions engaging in legal, policy, and healthcare work in support of currently and formerly incarcerated people. We recruited participants via direct outreach to those in our personal and professional networks and snowball sampling with the help of community organizations. We conducted semi-structured interviews and focus groups via Zoom. Formerly incarcerated participants were invited to choose their own code names for use in presentations and publications. Throughout the paper, we use only code names or descriptors of participant professional roles (e.g., *advocate* or *physician*) to identify participants. Interviews and focus groups lasted 1 to 1.5 h and were audio-recorded and transcribed verbatim, then analyzed using techniques based in grounded theory (Bryant & Charmaz, 2010; Corbin & Strauss, 2007). Both interview and focus group guides were developed to be intentionally open-ended. We sought to understand how decisions were made in the early days of the pandemic, with an initial question often being “tell us what you remember about when you first learned of the pandemic.” Focus group guides had questions about healthcare in prisons and priorities of advocacy work during the pandemic. Interview and focus group participants received a gift card as compensation for their involvement in the study.

Three researchers collaborated on data analysis for this project. To start, the authors read two to three transcripts independently and engaged in a process of open coding (Corbin & Strauss, 2007) to create an initial set of inductive codes grounded in the data and deductive codes based on prior research with similar populations (James, 2024; Jeske et al., 2023). We discussed our individual lists of codes and collaborated to develop a code list that would be adapted throughout our analysis. We then engaged in independent parallel coding of each transcript using the qualitative analysis software Atlas.ti. Reports were generated for each code, and associated quotations were then further refined into categories and later themes in an iterative process. These themes were discussed at length in research meetings. Our team is made up of a sociologist, a master’s prepared social science researcher, and students in sociology and medicine. We are diverse in terms of race and ethnicity and have varied connections to the criminal legal system, though none of us have lived inside a prison. This diversity, coupled with intentional reflexivity, allowed us to approach the data from many perspectives. This study was approved by the UCSF Institutional Review Board.

## THE CRISIS OF COVID-19 IN PRISONS

### Prisons as vectors for disease

Prisons have long been known to be devastating for the physical, mental, and social health of the people and communities they encounter. Overcrowding, poor ventilation, and congregate living contribute to a disproportionate prevalence of infectious diseases in prisons (Cloud et al., 2023). Community-based harm reduction resources such as needle exchanges are also limited in carceral settings, contributing to a significantly higher risk of disease from infectious, blood-borne pathogens like HIV and Hepatitis C (Hofmeister et al., 2018; Maruschak, 2023). Furthermore, dehumanization infiltrates nearly every interaction with prison staff, leading even the most benign attempts at health and risk mitigation to be met with skepticism, reluctance, and retaliation. When the COVID-19 pandemic emerged in early 2020, the California Department of Corrections and Rehabilitation (CDCR), like many government agencies, was unsure of how to act. Many of the public health strategies recommended by the Centers for Disease Control and deployed outside of carceral settings were incompatible with the prison environment or generated additional health harms to incarcerated people. For example, masking was a vital measure outside of prisons to contain disease and still allow individuals to work, seek care, and spend time away from home. In the carceral setting, masking could obscure the identity of residents inside and theoretically pose a security risk. Furthermore, social distancing in the carceral setting was impossible. While maintaining six feet of distance became the public health axiom outside, many over-crowded carceral facilities maintained standard distances of as close as three feet between beds (Kajstura & Landon, 2020).

Given these and other incompatibilities, many anticipated that COVID-19 could be a disaster for prisons. Healthcare providers and public health officials warned the public of the deadly combination of a violent, underprepared system known to cause and exacerbate illness and a devastating, rapidly-spreading virus. One healthcare provider who worked in a large state prison in the spring of 2020, acknowledged that the structural challenges of containing a virus in a prison setting put many healthcare professionals at a loss as to how to proceed:

What do you do with a population this size with a virus that’s this transmissible? How do we protect these guys? And so, every day you showed up and it seemed like every day… We didn’t know what we were doing, to be honest.

An advocate who supports people incarcerated in women’s prisons explained how prisons “are the most congregant settings imaginable,” and that those in power and organizers alike already knew that jails and prisons were “in a state of total public health crisis prior to a pandemic.” In reflecting on the transfer of 121 untested people to San Quentin State Prison in May of 2020, and the subsequent COVID outbreak that ultimately killed 29 people (“Judge Rules CDCR,” 2021; Sawyer, 2021; Williams & Griesbach, 2020), one advocate questioned:

What is going to happen when these institutions and buildings that were built in the 1800s and are operating at over 200% capacity in some areas of the prisons, when one CO [Corrections Officer] comes in sick?

Many across the state, including prison residents, advocates, and healthcare providers, knew the risks of porous institutions. With staff moving in and out of the facility each day, the risk of the virus coming in was high regardless of lockdowns or other measures initiated. This meant that many were anticipating the potential for an outbreak to occur at any moment.

### CDCR response to the crisis

In the spring of 2020, public health officials highlighted several strategies to limit the spread of COVID-19 in prisons and jails. The most urgent strategy was population reduction through the release of incarcerated people believed to pose “little or no public safety risk” (Barnert et al., 2020). In July of 2020, after the deadly COVID outbreak at San Quentin, California Governor Newsom committed to release 8,000 people by the end of August 2020 (Myers & Willon, 2020). Unfortunately, few people were released from CDCR through mechanisms like compassionate release. Targeted release of those deemed “high risk” for severe COVID morbidity and “low risk” of reoffending was slow and mismanaged. According to CDCR data from February 2021, of the 214 incarcerated people who died of COVID-19, their average age was 64, significantly younger than the average age of COVID deaths outside of prisons and jails. Additionally, 78% of those who died had a disability, and 72% were people of color, mirroring both the disparate impact of COVID-19 on marginalized communities outside of prisons and the vast overrepresentation of BIPOC and people with disabilities incarcerated in the United States. Tragically, 88% of those who died were considered at low risk of reoffending by CDCR (Rosen Bien Galvan and Grunfeld LLP, 2021) and thus should have been eligible for release by the governor’s own plan. Referring to a list of individuals who were deemed fit for release, one health provider noted:

We [CDCR] just couldn’t get it together… And I mean we had nine months. Like nine months. And the most horrifying part is, of the people, 11 [of my patients] that are dead were on that list. They shouldn’t have been there anymore.

In addition to population reduction through targeted release, public health officials urged carceral settings to screen for exposure and symptoms whenever transfers occurred, enact social distancing measures, educate residents and staff on COVID-19 transmission and prevention, provide resources for hygiene and disinfection strategies, and isolate those suspected of infection (Barnert et al., 2020). CDCR’s response to these recommendations included halting all visits, mandating a six-foot social distancing measure, and limiting the movement of incarcerated people outside of their cell walls. CDCR used lockdowns, a punitive measure often used to control the population, as a tool for COVID risk mitigation. Even threatening solitary confinement became a tool employed by prison staff to ensure compliance with institutional COVID rules (Song et al., 2023). This came with clear consequences for the health and well-being of the people experiencing being locked in their cells, often for 23 or more hours per day, for days, weeks, and even months on end:

There were months where these people were not really getting to leave their building. Like they were legit quarantined. And it’s not even that anybody in the building tested positive, it’s just everything was so locked down. They weren’t getting dayroom, they weren’t getting to go outside. Like normally they’d walk to chow or they’d have rec therapist groups outside on the yard. But they weren’t getting any of that for a couple of months there.

Despite the severe impact on the physical and mental health of many people, these lockdowns did little to mitigate the spread of disease, especially in older prisons without solid doors and sufficient ventilation (James et al., 2023). While health professionals did explain that the structural conditions of the prisons made the enactment of health-promoting behaviors impossible, the inconsistent and, in many ways, illogical enforcement of COVID policies on incarcerated people, but not on correctional staff, must also be acknowledged. While incarcerated people were forced to be locked in cells with limited access to resources, carceral staff moved into, out of, and between facilities freely. This discrepancy was acknowledged by both clinicians and residents, with one formerly incarcerated person describing, “As far as [prison staff] was concerned they wanted to keep you locked in. But why keep us locked in? You were the one that brought it in here.” Rather than allowing people outside where ventilation and space were possible, people were contained inside, in very tight quarters, and were unable to move freely and socialize. Instead, tools of the carceral setting designed for punishment, such as solitary confinement, a practice known to have devastating effects on physical and mental health (Cloud et al., 2020), were coopted for the medicalized implementation of quarantine and isolation. The carceral system attempted to prevent the introduction of an unknown virus by following public health guidelines that were clearly incongruent with its own purpose and physical limitations. The recognition of this dichotomy generated a significant amount of fear in incarcerated people, who had no choice but to find ways to protect themselves and their co-residents from harm.

### How are we going to keep ourselves safe?

Protecting oneself against disease is a constant part of prison life. Many formerly incarcerated people described the intense cleaning routines used in prison cells. In a context where they have almost no power over their surroundings, they do what they can to lower their risk. But COVID was particularly threatening.

As is now well understood, COVID risk mitigation necessitates both community commitment and infrastructural support for creating health and well-being. While many outside of prison have largely abandoned community and public health approaches to disease prevention, people in prisons have always engaged in community health practices, despite facing a carceral system that is antithetical to those practices. As described by several interviewed participants, people inside find ways to care collectively and to engage in public and community health, despite real and perceived threats, as a form of healing, caring, and, importantly, resistance. These methods of care, which became severely limited and even punishable during the pandemic, included both informal interactions like talking with friends, sharing food, and offering support with activities of daily living, and formal group programming that provided people with educational opportunities, trauma-focused mentorship, and addiction recovery. Through both the loss of these existing systems and their adaptation to fit an evolving and uncertain climate, the pandemic put a spotlight on the carework done by incarcerated people to resist the health-related harms imposed on them by the carceral system and its policies.

### CAREWORK AS RESISTANCE

Joy James and Jaime Amparo Alves describe the “Captive Maternal” as “the ungendered but often racialized female, whose function and labor provide emotional and material support that furthers communal and familial stability” (James & Alves, States of Security, Democracy’s Sanctuary, and Captive Maternals in Brazil and the United States, 2018). The carework provided by this figure is an often-invisible labor that, in the context of varying levels of violence and trauma inflicted by the state, functions to provide a “foundation for sanctuaries resisting repression” (Doughty et al., 2023). As further described by James and Alves, “Maternal rage becomes the political resource for the dispossessed; the captive Black maternal (ungendered) refuses death and defeat despite the inevitability of each” (James & Alves, States of Security, Democracy’s Sanctuary, and Captive Maternals in Brazil and the United States, 2018). The ungendering of the Captive Maternal reflects the inclusion of not only cisgender women, but also those “feminized” into caregiving. Although the prison system as an institution simultaneously strips gender away and imposes gender norms on its residents, the Captive Maternal is a role that incarcerated people of all genders have always had to play as they face the medical neglect and violence brought on by a punitive system functioning in a healthcare capacity. Providing emotional and material support to each other, as James suggests, is what allows incarcerated people to “refuse death and defeat” within a system designed to repress. As we will describe below, carework provided for incarcerated people by incarcerated people was not new when COVID began to enter prisons. Peer-led support and advocacy groups have, for decades, provided emotional and material support to incarcerated people. This work has been both invisible and yet deeply relied upon by the prison system.

Birdy is a White woman in her 70s. She is a former nurse who had resided for decades in the honor unit, a “privileged” dormitory for individuals without disciplinary infractions (Campbell, 2010). She told us that as an older White woman and former working professional who had been living an upper-middle-class life prior to incarceration, she had relative privilege within the prison. She was offered leadership opportunities not always made available to women of color and those with less education, and she tried to use that privilege to benefit others inside. Birdy described to us the long-termers organization she led while incarcerated, prior to the onset of the pandemic. This group supported people sentenced to more than 10 years to “survive doing a long term in the prison system and…. keep the population up on changing laws, what sort of opportunities would be available for people.” She was also a leader for the Golden Girls group, an advocacy and support organization for women over 55 inside. They ensured their institution took measures to provide accommodations to older women and prevent heat prostration given a lack of central cooling.

An RN by training, Birdy also utilized her unique skillset and knowledge of pertinent medical terminology to directly support other residents facing healthcare concerns. She would act as a first responder, helping carceral staff relay critical information to the medical response team during emergencies. Having built a rapport with the housing staff, Birdy would ensure that truly emergent medical needs would be taken seriously:

They would sort of rely on me, because the way the system goes, the custody staff would walk down and look at the potential patient and then they would relay that information to the medical staff. So, I would give them words to say. I would say, ‘skin is cold and clammy, face is flushed’ etc. And that in their mind would help to make certain that the medical staff would actually respond.

Utilizing her knowledge of the *Coleman* and *Plata* class action cases, which recognized the constitutionally inadequate medical and mental health care provided in California prisons (Brown v. Plata, 2011, 563), Birdy also became known for helping others write grievances when their healthcare was inadequate or negligent:

I had set up systems and I kind of became known to help others write grievances when things weren’t going their way… ‘I need healthcare that is commensurate with community standards.’ That was a phrase I used all the time. Because that’s what the Coleman/Plata lawsuit says. So that worked.

Filing grievances for staff misconduct, sexual abuse, harassment, discrimination, or healthcare-related misconduct within CDCR involves submitting a form within 60 days of the event, interpreting 10 possible grievance outcomes, and, if necessary, appealing CDCR responses. Residents can be labeled as abusing the grievance system should more than one health care grievance be submitted within 14 calendar days, resulting in restrictions on filing rights. Having specific advocates who understood how to navigate this process and use appropriate language gave individuals hope that their claims would not go unaddressed.

Prison hospice programs, staffed by trained volunteers of incarcerated peers, address another critical area of carceral neglect and allow space for compassion and care that otherwise contradicts the status quo (Sharp, 2022). One formerly incarcerated woman, Katie, described starting a “Compassionate Companions Program” that would maintain a vigil for patients who were terminally ill inside. These programs are necessary because of the extreme sentences levied in the US and limited options or possibility for release. Between 2015 and 2021 in California, 95 out of 306 incarcerated people who were referred for compassionate release died before their application for the process was processed (Chi-Fang Tsai, 2014; Office of Assemblymember Phil Ting, 2022). Programs like Compassionate Companions, developed by people inside, ensured that no one was left alone while they were dying.

The ability to care for each other was often subversive or done only at the discretion of an approving carceral officer. Sharing food or medication, physical touch, and even sending messages to other yards of the prison are all violations that can lead to write-ups or worse. While groups like the Golden Girls and Compassionate Companions have fought to be allowed to do formal and informal carework, this is not automatic and equally applied. One woman, who asked that we call her Second Chance, described how her inside family cared for her after she had her gallbladder removed, describing, “[the COs] let them come in my room and they would bathe me and help me and take care of me.” She was fortunate that the staff allowed this practice and grateful to receive support as she recovered from surgery inside (James, A Second Chance at Health, 2021).

As described, incarcerated people have historically engaged in multimodal forms of carework to survive the prison system. Though it is not often acknowledged as an essential piece of the public health infrastructure within prisons, peer-to-peer carework is relied upon as a safety net for residents who would otherwise continue to be mistreated, neglected, and retaliated against by the carceral system. Expanding upon Doughty et al. (2023) suggestion that those on the outside who cared for their incarcerated kin during the pandemic embody Joy James’ Captive Maternal, we acknowledge the radical carework by and for incarcerated people as reflective of this same ideology (James & Alves, States of Security, Democracy’s Sanctuary, and Captive Maternals in Brazil and the United States, 2018). The COVID pandemic not only illuminated the role of incarcerated people as Captive Maternals, but also, as we will explore, revealed the creativity and resilience required to resist an even greater threat to isolation and disposability.

## METHODS OF CAREWORK DURING COVID

The responsibility of CDCR to provide adequate resources for hygiene and overall health posed a challenge for prisons facing pandemic-related staff and supply shortages. A lack of carceral staffing and lockdown measures meant that many of the resources that incarcerated people needed to stay healthy, including access to adequate food, laundry, and disinfectants, were not provided. As one woman, who asked us to call her Freedom, who was quarantined in the early days of the pandemic, described:

The room was hot. They brought our food to the room. The food was some kind of like slop. Sometimes we didn’t eat it…. My high blood pressure had went up [*sic*]. I got anxiety from being in a room. It was just very uncomfortable during this time. It really wasn’t clean. Like it was supposed to have been cleaned, giving us any supplies to clean our rooms, to disinfect our rooms. Bleach and things like that. That didn’t come about. We had to use what we had…. we had to use shampoo and our own little personal items to keep our room clean and to keep me and my bunkies safe. So going out to the shower we made our own concoction for chemicals to spray. Like spray the handles before we took a shower so we wouldn’t catch anything. Spray the doorhandles. Things was [*sic*] very unclean there for a while.

The lack of access to resources needed to maintain one’s health and safety extended beyond the material to include disruptions of established forms of carework, especially support groups. With many people locked in their cells for days, weeks, or months on end, and a relative lack of access to mental health services, many people served as the sole sources of support for their cellmates (James et al., 2023). While COVID disrupted some mechanisms of care, it presented an urgent need for new models of support to be imagined. One outside organizer, who has spent decades advocating on behalf of incarcerated women, noted that carework is not traditionally thought of as organizing and is not often visible as work, but that due to the pandemic, “there’s been a lot more space opened up for conditions that impact people’s health being actually taken really seriously.” The COVID-19 pandemic provided the outside world with an opportunity to recognize carework done inside as a form of resistance against state violence. As the organizer described, COVID-19 laid bare the ways in which “people take care of each other inside and the ways that those relationships are not necessarily recognized as forms of resistance.”

### Individual advocacy and resistance

When it became clear that CDCR was not going to protect its residents from the incoming onslaught of COVID-19, Birdy began engaging in acts of advocacy, resistance, and carework. She wrote letters to the shift leader, the warden, and the medical director asking about COVID policies, requesting additional cleaning materials and ventilation, and making sure they were following the emerging and evolving public health guidance. She knew she was at risk as an older woman, but she was even more concerned for the many people around her with chronic conditions. While much of prison life had shut down, people were still moving between and across units cleaning (including cleaning the rooms of people who had tested positive for COVID), delivering food, and working for a few cents an hour sewing masks (considered contraband for people incarcerated at CDCR) that would be sold and distributed across the state.

While many carceral staff members refused to wear masks, incarcerated people were punished for trying to do so. They were forbidden from wearing what was quickly becoming mandated personal protective equipment outside of prisons based on the purported “security” risk masks posed inside. We received reports of people being beaten by guards for wearing masks in men’s prisons. Some women described write-ups and other disciplinary infractions. Birdy saw this happening, but she was following the science as much as she could from inside. She and others started to make masks out of rubber bands and scrap materials—just as many on the outside did in the early days of the pandemic when PPE was not readily available. Birdy described how she “proudly wore” her mask and “dared” anyone to tell her she couldn’t—something she felt she could do as an older White woman within the institution. Within a couple of weeks, not only were other residents defying orders and wearing their masks, but even staff were eventually required to do so. Birdy used her relative privilege to engage in acts of resistance against a harmful set of policies she knew could kill her and her friends inside.

### Finding new ways to support each other inside

Before CDCR policies forced people to be locked in their rooms for most of the day, incarcerated people were already showing a transformed collective consciousness that prioritized group health and safety. One participant recalled a situation where someone in the honor unit was accused of having a fever and not disclosing it to the rest of the population. She described an intervention where “a whole group of us got on that person and said, look, it is not okay to keep quiet about these things. You put everybody at risk.… It was handled comfortably, eventually… But the reaction of the unit was very clear.” Vital to this example is the recognition that to survive the pandemic, people had to create mutual understanding and engage in direct intervention without the involvement of the institution.

When direct communication became limited by a lack of mobility across housing units, incarcerated women found a variety of ways to use their knowledge, experience, and social capital to improve their circumstances. One organizer described someone who was “facilitating processes of letter writing and note passing inside to ensure that there [was] up-to-date information about what’s happening in the [prison] skilled nursing facilities…” With lockdowns and lack of visitation, this organizer noted that the pandemic was a time of increased isolation. Sharing organizing information—or even just being in communication with each other to check in on friends and loved ones—was incredibly difficult. Yet, those inside found creative ways to stay in touch.

Another organizer noted how “powerful” it was to watch how people find ways to care for and organize with each other.

The ways in which folks have combated the isolation that’s obviously happening… some of the creative ways that people are finding to communicate with one another and continue providing for one another. There have been more instances where folks are communicating via sending an email to someone to check in on someone else or to relay information in ways that just speaks to the incredible infrastructure and organizing structures that already existed. The fact that that can translate and happen so quickly when people are isolated and prevented from communicating with one another.

### Organizing with people outside

Those living inside prisons were also doing extensive organizing work with people outside to advocate for the implementation of public health guidelines and bring accountability to the prison industrial system that often prioritized surveillance over risk mitigation strategies. Relaying stories from the inside was a critical form of carework for incarcerated people; bringing attention to the conditions inside was often the only way to change those conditions and get help for those most in need. By directly broadcasting their experiences to the outside world through letter writing and partnering with advocates, they were able to garner the support of outside organizers, and place CDCR in a position to have to speak to their policies and procedures.

Furthermore, the more widespread use of video communication platforms during the pandemic allowed for a transformation of how organizing could be done with those living inside prisons. Now, incarcerated people could use the prison phone system to call into organizing meetings and press conferences. Because these events were happening over Zoom, their grievances could easily be heard by hundreds of people in attendance. This allowed the voices of currently incarcerated people to be centered in organizing in ways that had been more challenging when meetings and events happened primarily in person.

Social media also became a medium for currently incarcerated people to co-construct a narrative with organizers about what was happening inside, sparking the attention of CDCR and the governor:

But here was media coming out… where currently incarcerated people were driving the narrative, and the CDCR was in the counter narrative section of the article. Like they had to respond and then be rebutted. And so, like it was, for the first time, and I think like it was largely because of the existing relationships [between organizers and incarcerated people], currently incarcerated people were driving the narrative and CDCR was having to respond. And even, at different times, the governor had to respond to what they were saying.

These platforms also became sources for fundraising. One consequence of lockdown policies was that some individuals inside couldn’t work (what work was considered “essential” during the pandemic shifted across the early months), and many families were not able to contribute to the cost of canteen goods or phone calls/emails. Prison canteens, also known as commissaries, provide necessary supplementation to the inadequate hygiene products and food available behind bars, while also serving as a source of comfort and connection to the outside world. It is through contribution to one’s canteen purchases that families can support the health and well-being of their loved ones inside (Rege-Rex, 2023). As an organizer explained:

And then we also recognized the need for the people to go to the store to get canteen because, as we know, as people might’ve seen on a lot of social media posts, these meals from prison are just horrendous. They are not conducive to a healthy immune system. And also too, there’s just a lack of supplies and soap. So, we started a campaign on Facebook [to raise funds].

The centering of the voices of currently incarcerated people was critical to the engagement of outside organizers during COVID. In fact, the ability to maintain direct and consistent communication with people inside encouraged increasing numbers of formerly incarcerated people to engage in organizing and advocacy work. Formerly incarcerated people expressed feeling committed to helping those on the inside, speaking to this strong sense of community and caregiving that occurs in the carceral system even after an individual is released. One organizer, who was recently released, also described this:

And what I noticed my last couple of years was a lot of formerly incarcerated people going into spaces around reentry and things that were more directly related to the struggles that they were having, which you know totally makes sense, but it made me feel that you have to intentionally continue to stay connected with *currently* incarcerated people and take their lead and their direction in order to have that perspective and to keep the values aligned with trying to get people home…

In addition to highlighting the needs and experiences of incarcerated people to resist COVID-19, this centering of the narratives of incarcerated people provided another organizer with the opportunity to shed light on the crises that are always present inside prisons:

Like we were able to collectively, with people inside, push some around the suicide crisis and COVID. But I think one of the tools we have used to kind of force that attention is using social media to blast CDCR for horrible conditions. Like no water, no medical, rats in quarantine, that kind of stuff at CIW. And then using that publicity to get more mainstream attention from the media.

## CONCLUSION

Through our digital ethnography of the COVID-19 pandemic in California and interviews with people directly impacted by COVID-19 in prison, we came to see that, in the context of women’s prisons, carework and resistance operate hand in hand. Care is a critical form of resistance in a punitive system, and resistance, fighting for change in policy and treatment, is an essential component of how people inside care for each other. This form of care, which some refer to as “fierce care” (Callahan & Paradise, 2017) or “radical care,” like other forms of feminized work, is often deemphasized and not recognized as a critical tool of resistance. One organizer expressed frustration that “that type of organizing isn’t seen or talked about.” Protests, boycotts, hunger strikes, and riots are often the main representations of prison resistance that we see highlighted in the media (Gatewood & Norris, 2019). While these are all critical tools of prison resistance, they are only the tip of the iceberg of the organizing work being done behind and beyond prison walls. Avenues such as legal action, filing grievances, art, journaling, support groups, forging connections with outside groups, and education are all central forms of resistance (Gatewood & Norris, 2019; Law, 2009). As we have argued, carework should also be considered within the constellation of resistance activities occurring everyday behind bars. While carework in prisons has always occurred through the formation of peer-led support groups and the individual efforts of residents acting as advocates for others, the pandemic brought a wave of challenges that forced residents to adopt new ways of caring for each other. On the inside, residents engaged in direct acts of resistance and supported each other in submitting grievances to advocate for greater protection from viral transmission. When collaborating with outside organizers, the adoption of new technology allowed for the centering of narratives of incarcerated people directly.

As we saw in the context of the COVID-19 pandemic, finding ways to support and care for each other inside is an essential component of resistance in women’s prisons. Advocates and organizers we spoke with emphasized the “creative ways people are organizing to support each other” in the face of a crisis and heightened separation. Centering and naming this as carework, as labor, and as resistance, is in and of itself a radical, feminist act. The Captive Maternal, serving as an anchor for community-based revolutionary change, remains a consistent and adaptive care provider that gives hope for survival amid the dual harms of a pandemic and incarceration. And yet, she remains largely invisible. Even in our study, conducted from an explicitly Black feminist lens with a focus on racial justice, we saw that the stories of White women often became central given who is released from prisons and jails (vs. who remains trapped inside), who is more likely to be connected to advocacy groups and organizations, and who felt the relative safety to boldly start advocacy campaigns during incarceration. This is both a limitation of our own analysis and a further commentary on how racism is perpetuated in prisons. Birdy was able to wear a mask as a form of protest of the public health policies; others described watching someone being beaten by guards for taking a similar action. However, we again emphasize that we saw quiet forms of carework and resistance taken up by Black and Brown women. Sharing food, medicine, or information; being a listening ear to someone in crisis; writing to comrades outside to share details of policy changes inside: all are forms of carework and all are forms of resistance inside.

Although the pandemic provided us with a valuable lens into the ways that incarcerated people resist death in prison, we must recognize this as just an example of the ongoing public health efforts residents engage in to survive in a system that relies on dehumanization to fulfill its punishment purpose. Carework is essential work provided by and for the residents of the carceral system and should be recognized as such. People who are incarcerated have led efforts to improve health and well-being inside and, through radical acts of care, are doing the work to help us imagine alternative models of care and resistance and, ultimately, a world without prisons.
